# Prevalence and phylogenetic analysis of haemoplasmas from cats infected with multiple species

**DOI:** 10.1016/j.mimet.2014.10.013

**Published:** 2014-12

**Authors:** Larissa Campos Aquino, Chelsea A.E. Hicks, Marcela C. Scalon, Maíra G. da M. Lima, Marcelle dos S. Lemos, Giane Regina Paludo, Chris R. Helps, Séverine Tasker

**Affiliations:** a Laboratory of Veterinary Clinical Pathology, College of Agronomy and Veterinary Medicine, University of Brasília, Campus Universitário Darcy Ribeiro, Brasília 709010-900, Brazil; b School of Veterinary Sciences, University of Bristol, Langford House, Langford, Bristol BS40 5DU, UK; c Molecular Diagnostic Unit, Langford Veterinary Services, Langford, Bristol BS40 5DU, UK

**Keywords:** Haemoplasmas, Brazil, Cats, Prevalence, Co-infections

## Abstract

*Mycoplasma haemofelis* (Mhf), ‘*Candidatus* Mycoplasma haemominutum’ (CMhm) and ‘*Candidatus* Mycoplasma turicensis’ (CMt) are agents of feline haemoplasmosis and can induce anaemia in cats. This study aimed to determine the prevalence and phylogeny of haemoplasma species in cats from Brazil's capital and surrounding areas, and whether correlation with haematological abnormalities existed. Feline haemoplasmas were found in 13.8% of 432 cats. CMhm was the most prevalent species (in 13.8% of cats), followed by Mhf (11.1%) and CMt (4.4%). Over 80% of haemoplasma-infected cats harboured two or more feline haemoplasma species: 7.1% of cats were co-infected with Mhf/CMhm, 0.4% with CMhm/CMt and 3.9% with Mhf/CMhm/CMt. Male gender was significantly associated with haemoplasma infections. No association was found between qPCR haemoplasma status and haematological variables, however CMhm relative copy numbers were correlated with red blood cell (RBC) numbers and packed cell volume (PCV). Haemoplasma 16S rRNA gene sequences (> 1 Kb) were derived from co-infected cats using novel haemoplasma species-specific primers. This allowed 16S rRNA gene sequences to be obtained despite the high level of co-infection, which precluded the use of universal 16S rRNA gene primers. Within each species, the Mhf, CMhm and CMt sequences showed > 99.8%, > 98.5% and > 98.8% identity, respectively. The Mhf, CMhm and CMt sequences showed > 99.2%, > 98.4% and > 97.8% identity, respectively, with GenBank sequences. Phylogenetic analysis showed all Mhf sequences to reside in a single clade, whereas the CMhm and CMt sequences each grouped into three distinct subclades. These phylogeny findings suggest the existence of different CMhm and CMt strains.

## Highlights

• Over 80% of haemoplasma-infected cats had more than one haemoplasma species.
• The use of species-specific primers allowed derivation of 16S rDNA sequences from co-infected cats.
• Male gender was significantly associated with haemoplasma infection.
• Phylogenetic analysis showed subclade formation within two of the haemoplasma species sequences.

## 1 Introduction

Haemoplasmas are haemotropic mycoplasmas lacking a cell wall that attach and grow on the surface of red blood cells and can cause infectious anaemia in different mammalian species. Although some of their basic characteristics are known (Messick, 2004), they have not yet been successfully cultivated in vitro. Three main haemoplasma species can infect cats: *Mycoplasma haemofelis* (Mhf), ‘*Candidatus* Mycoplasma haemominutum’ (CMhm) and ‘*Candidatus* Mycoplasma turicensis’ (CMt). Mhf is the most pathogenic, often leading to haemolytic anaemia during acute infection. In contrast, CMhm and CMt are less pathogenic, but when combined with Mhf or retrovirus infection may also induce anaemia (Tasker et al., 2009).

Haemoplasmas are found throughout the world and have previously been identified in cats (Biondo et al., 2009a), dogs (Biondo et al., 2009a), cattle (Girotto et al., 2012), capybaras (Vieira et al., 2009), lions (Guimaraes et al., 2007) and deer (Grazziotin et al., 2011) in Brazil. Recently, the zoonotic potential of haemoplasmas has been reported after the molecular identification of haemoplasmas in immunosuppressed humans and professionals, some with frequent exposure to haemoplasma infected animals (dos Santos et al., 2008, Steer et al., 2011, Sykes et al., 2010). Moreover, domestic cats may act as a source of haemoplasma infection for wild animals (André et al., 2014).

Previous studies have reported the prevalence of haemoplasmas in domestic cats from different Brazilian states such as Mato Grosso do Sul (MS) (36.4%) (Santis et al., 2014), Rio de Janeiro (RJ) (12%) (Macieira et al., 2008), Rio Grande do Sul (RS) (21.3%) (Santos et al., 2009), Maranhão (MA) (12%) (Braga et al., 2012), São Paulo (SP) (32% and 6.5%) (André et al., 2014, de Bortoli et al., 2012) and Mato Grosso (MT) (8.4%) (Miceli et al., 2013). However these studies have not consistently reported haematological findings or phylogenetic analysis. In those that have evaluated phylogeny, only short (less than 600 bp) 16S rRNA gene sequences have been used (André et al., 2014, Miceli et al., 2013, Santis et al., 2014) or sequences were not submitted to GenBank (Braga et al., 2012). Additionally, few positive cats and no more than two reference sequences for each haemoplasma species were used in phylogenetic analysis. Further work on the phylogeny of Brazilian feline haemoplasmas is required, using (near) complete 16S rRNA and other genes if possible. The aim of this study was to assess the prevalence and phylogeny of haemoplasmas from naturally infected cats in Brazil's capital, Brasília, and surrounding areas, and to determine whether any correlation existed between haemoplasma infection and haematological abnormalities.

## 2 Materials and methods

### 2.1 Sample collection

From 2009 to 2013, EDTA-anticoagulated feline blood samples were obtained from the following groups in order to acquire as large and diverse a population of samples as possible: i) cats from Brazil's capital, Brasília, and surrounding areas that attended the local veterinary teaching hospital or private clinics, ii) owned cats from 7 cities in the surrounding areas seen during the 2012 and 2013 anti-rabies vaccination campaign, iii) owned cats from one city village outside Brasília sampled during a leishmaniasis surveillance programme conducted by the public health service, and iv) feral cats from a shelter located in Brasília's surrounding area.

### 2.2 Ethics Approval

Data regarding gender were available but not health status or age. The project was approved by the University of Brasília (UnB) ethics committee under the protocol number UnBDOC no. 43938/2012.

### 2.3 Haematological analysis

Haemoglobin concentration and red blood cell (RBC) numbers were determined using a semi-automatic veterinary blood cell counter (ABC Vet-Horiba® ABX diagnostics, Brazil) and the packed cell volume (PCV) was determined by microhaematocrit centrifugation. Mean corpuscular volume (MCV) and mean corpuscular haemoglobin concentration (MCHC) were calculated from haemoglobin, PCV and red blood cell count. Anaemia was defined as a PCV < 24%.

### 2.4 DNA extraction

DNA extraction from 100 μL of blood was performed using a commercial kit (Illustra blood genomicPrep Mini Spin Kit, GE Healthcare, UK) according to the manufacturer's instructions. The DNA was eluted with 100 μL of elution buffer provided with the commercial kit and stored at − 20 °C prior to analysis.

### 2.5 Control and generic haemoplasma conventional PCR assays

In order to check for the presence of PCR inhibitors and evaluate the DNA quality, all samples were subjected to a control conventional PCR to amplify a fragment of the glyceraldehyde-3-phosphate dehydrogenase (GAPDH) gene using primers described by Birkenheuer et al. (2003), as part of another previous study assessing DNA extraction efficiency. The protocol for this PCR included the following: 1 μL of DNA template was amplified using 0.5 μM of each primer, 1.25 U *Taq* DNA Polymerase recombinant, 0.2 mM dNTPs, 1× PCR buffer and 1.5 mM MgCl_2_ (all Invitrogen™), with water to 25 μL. Amplification was performed in a thermal-cycler (FTGene5D, Techgene, UK) with an initial denaturation step of 94 °C for 5 min, followed by 40 cycles of denaturation (94 °C; 30 s), annealing (52 °C; 1 min), extension (72 °C; 1 min) and a final extension at 72 °C for 5 min. Water was used as a negative control and DNA extracted from non-infected cat blood as a positive control. Samples giving bands of 400 bp on a 1.5% agarose gel stained with ethidium bromide were considered positive and, therefore, had DNA of sufficient quantity and quality for further molecular analysis.

Following GAPDH conventional PCR, DNA samples were then screened for haemoplasma infection using a generic haemoplasma conventional PCR adapted from Criado-Fornelio et al. (2003) to amplify 595 bp fragments of the 16S rRNA gene. In the reaction, 2 μL of DNA template was amplified using 0.5 μM of each primer, 1.0 U *Taq* DNA Polymerase recombinant, 0.25 mM dNTPs, 1× PCR buffer and 2.5 mM MgCl_2_ (all Invitrogen™), with water to 25 μL. Amplification was performed in a thermal-cycler (FTGene5D, Techgene, UK) with an initial denaturation step of 94 °C for 10 min, followed by 40 cycles of denaturation (94 °C; 30 s), annealing (50 °C; 30 s), extension (72 °C; 30 s) and a final extension at 72 °C for 10 min. Water was used as negative control and DNA from a naturally infected haemoplasma cat previously diagnosed by cytology and PCR (species not defined) was used as a positive control. PCR products underwent electrophoresis in a 1.5% agarose gel stained with ethidium bromide, and samples with fragments of 595 bp were identified as positive.

### 2.6 Haemoplasma species-specific real-time quantitative PCR assays and relative copy number determination

All of the DNA samples generating positive results by the conventional generic PCR were shipped to the University of Bristol, UK for subsequent real-time quantitative PCR (qPCR) as previously described (Peters et al., 2008b) to identify the haemoplasma species (Mhf, CMhm and/or CMt) present and determine relative copy numbers. All qPCR reactions were duplexed with an internal control (feline 28S rRNA gene) assay to demonstrate the presence of amplifiable DNA and the absence of PCR inhibitors; a threshold cycle (Ct) value of 22 was used as a cut off. In each run, DNA from known haemoplasma positive cats was used as a positive control and water as a negative control.

Relative copy numbers were calculated using the *E*^ΔCt^ equation, with the assumption that the highest Ct in the sample set equalled 1 haemoplasma copy/reaction (ΔCt = highest Ct − sample Ct) and taking into account the Mhf, CMhm and CMt qPCR reaction efficiencies (*E*) previously determined (Peters et al., 2008b) from standard curves.

### 2.7 16S rRNA gene sequencing

The 11 CMt, 10 Mhf and 10 CMhm positive samples representing different geographical locations with the lowest Ct values were chosen for 16S rRNA gene amplification and sequencing. However, in order to sequence the 16S rRNA gene of individual haemoplasma species from the many cats with haemoplasma co-infections, species-specific 16S rRNA gene primers were designed to amplify around 1200 bp of the 16S rRNA gene through a conventional PCR. Briefly, complete 16S rRNA gene sequences from feline haemoplasma species available on GenBank (accession numbers AY831867, DQ157150, DQ157151, DQ464417, DQ464418, DQ464419, DQ464420, DQ464421, DQ464422, DQ464423, DQ464424, DQ464425, AF271154, AY150974, AY150978, AY150979, AY150980, AY150981, U88564, AF178677, AF548631, AY069948, AY150972, AY150976, AY150977, AY150984, AY150985, U88563, U95297) were downloaded and aligned using Clustal-W on MacVector ver 13.0.3. The consensus sequence generated for each of the three feline haemoplasma species was used to manually identify the most specific regions of the 16S rRNA gene to which species specific primer pairs could be designed. Short sequences ranging from 25 to 30 bp were selected as forward and reverse primers and tested using Primer3 (web version 4.0.0; http://primer3.ut.ee/) against the consensus sequence to obtain primer pairs with suitable melting temperatures (Tm), self-complementarity, 3′ self-complementarity and hairpin formation.

Two primer pairs for each feline haemoplasma species were selected for evaluation based on their predicted product size, Tm and minimal pair complementarity (Table 1). The primer specificities were first tested by BLAST searching against all existing DNA sequences stored in GenBank and subsequently each primer pair was subjected to PCR with a template of as high relative copy number as possible available (10^7^ of CMhm, 10^7^ of Mhf or 10^4^ of CMt) of their non-target feline haemoplasma species to evaluate cross-reactivity. The selected primers were tested in a 25 μL reaction containing 12.5 μL of 2× Promega GoTaq® Hot Start Colorless Master Mix (USA) with 0.2 μM of each primer and 1 μL of DNA template, to a final volume of 25 μL with water. The reaction was performed on a SureCycler 8800 thermal cycler (Agilent Technologies, USA) with cycling conditions as follows: 95 °C for 5 min, followed by 45 cycles of amplification (95 °C, 10 s; 62 °C, 30 s; 72 °C 90 s) with final extension of 72 °C for 5 min. Cats singly infected with Mhf, CMhm and CMt, previously identified by the species-specific qPCR assays and water, were used as positive and negative controls, respectively. Products were identified by electrophoresis in a 1.5% agarose gel stained with ethidium bromide.

**Table 1:** 16S rRNA gene primer sequences designed for haemoplasma species-specific conventional PCR amplification of haemoplasma DNA from positive cats for the 16S rRNA gene sequencing.

Primer name	Primer sequence	Tm^b^ (°C)	Product size (bp)
MhfFw1	GCTGATGGTATGCCTAATACATGC	59	1336
MhfRev1	GCCCACTCCTCTCATAGTTTGA		
MhfFw2^a^	CGAACGGACTTTGGTTTCGG	59	1214
MhfRev2^a^	CTTCAAGGAGGCGAATTGCAG		
CMhmFw1	TACTCTCTTAGTGGCGAACGG	60	1314
CMhmRev1	CTCCCATAGTTTGACGGGCG		
CMhmFw2^a^	AGGGTTTACTCTCTTAGTGGCG	59	1375
CMhmRev2^a^	TCCAGTCAAAATTACCAATCTAGACG		
CMtFw1	CTGTCCAAAAGGCAGTTAGCG	59	1312
CMtRev1	TGTGTTTTCAAATGCCCCTTCC		
CMtFw2^a^	GTCCTATAGTATCCTCCATCAGACAG	59	1039
CMtRev2^a^	CGACACATTGTACTCACCATTGTAA		
Internal forward	GGGATTAGATACCCCAGTAGTCCAC	59	600
Internal reverse	GTGGACTACTGGGGTATCTAATCCC		

a Primers selected as external primers for sequencing.

b Tm = melting temperature.

PCR products of the expected ~ 1200 bp size were purified with the NucleoSpin® Gel and PCR Clean-up kit (MACHEREY NAGEL GmbH & Co.) according to the manufacturer's instructions, quantified with a Qubit™ fluorometer (Invitrogen™) and submitted to the DNA Sequencing & Service (MRCPPU, College of Life Sciences, University of Dundee, Scotland, www.dnaseq.co.uk) for sequencing using an Applied Biosystems Big-Dye Ver 3.1 chemistry on an Applied Biosystems model 3730 automated capillary DNA sequencer. Sequencing was performed in the sense and antisense directions using the external species-specific primers (MhfFw2/MhfRev2, CMhmFw2/CMhmRev2, CMtFw2/CMtRev2) and internal primers (Table 1). The internal primers were manually designed in highly conserved regions of the 16S rRNA gene after alignment of 16S rRNA gene sequences from the three haemoplasmas species and checked using Primer3.

### 2.8 Phylogenetic analysis

Sequence editing and analysis were performed in MacVector v13.0.3, Inc. Reconstruction of near-complete 16S rRNA gene sequences for the 11 CMt, 10 Mhf and 10 CMhm positive samples was performed by combining individual sequences derived from the external and internal primers. These newly derived 16S rRNA gene sequences and the 16S rRNA gene haemoplasma species sequences available from GenBank were then aligned using Clustal-W. The phylogenetic tree was generated using the Neighbor-Joining method from a P-distance matrix and with data resampled 1000 times to estimate the confidence of branching patterns.

The novel haemoplasma 16S rRNA gene sequences generated in this study were submitted to GenBank under the following nucleotide accession numbers: Mhf sequences (KM275238–KM275247), CMhm sequences (KM275248–KM275257) and CMt sequences (KM275258–KM275268).

### 2.9 Statistics

In order to accurately determine the prevalence of haemoplasmas, the sample size required was estimated according to Thrusfield (1986) and based on prior evidence (Barker et al., 2010, Biondo et al., 2009b) with precision of 5% and 95% confidence intervals.

Data were entered into Excel® and statistical evaluation was carried out using SPSS for Windows (SPSS Inc., Chicago IL, USA). Categorical variables (gender, conventional PCR and qPCR status) prevalence values were calculated together with 95% confidence intervals using the Clopper–Pearson exact binomial method and differences were analysed by *X*^2^ test. Cats were divided into five groups according to haemoplasma status based on the qPCR qualitative results generated: haemoplasma negative, CMhm only infected, CMhm and Mhf co-infected, CMhm and CMt co-infected, and CMhm, Mhf and CMt co-infected (no Mhf only infected or CMt only infected cats were found). The Kolmogorov–Smirnov test was used to test for normal distribution of continuous variables (PCV, RBC, haemoglobin concentration, MCV and MCHC). Kruskal–Wallis testing was used to determine if there were any significant differences among the haemoplasma status groups for non-normally distributed continuous haematological variables. Spearman's test was used to assess correlation in non-normally distributed data. Significance was assigned as a p value < 0.05.

## 3 Results

It was estimated that a sample size of 349 cats was needed to reliably determine the prevalence of feline haemoplasmas in Brasília and surrounding areas. From 2009 to 2013, 451 cats were sampled.

Generic haemoplasma conventional PCR and haemoplasma species-specific qPCR results are shown in Table 2. All 451 samples were positive for GAPDH DNA by conventional PCR. Of the 451 cats sampled, 80 (17.7%) were haemoplasma positive by the generic haemoplasma PCR. However, due to sample volume limitations, only 61 of these 80 haemoplasma positive samples could be subsequently subjected to haemoplasma species-specific qPCR. Thus 432 cats comprised the final study population. Gender data were not available for 70 cats; of the remaining 362 cats, 176 (48.62%) were female and 186 (51.38%) were male. All 61 qPCR-tested samples were positive for 28S rRNA gene at a Ct of ≤ 22. Sixty of the 61 samples were haemoplasma positive for any species by qPCR, representing an overall prevalence of haemoplasma infection of 13.8% in the 432 cats. Only one cat was positive in the generic haemoplasma PCR but not in any of the haemoplasma species-specific qPCRs. Of the 432 cats, 48 were positive for Mhf, 60 for CMhm and 19 for CMt, representing overall prevalences of 11.1%, 13.8% and 4.4%, respectively for each haemoplasma species. The CMt prevalence was significantly lower (p = 0.0002) than that of CMhm and Mhf, but no significant difference was observed between the prevalences of CMhm and Mhf (Table 2). There was a very high proportion of cats with dual or triple haemoplasma co-infections, with 50 out of the 60 (83%) haemoplasma infected cats being infected with more than one species, and no cats were solely infected with either Mhf or CMt. Of 432 cats, 17 (3.9%) were triple infected with Mhf, CMhm and CMt, 31 (7.1%) were dual infected with Mhf and CMhm, and two cats (0.4%) were dual infected with CMhm and CMt. All of the negative and positive controls gave appropriate results in all PCRs.

**Table 2:** Number and percentage of cats generating positive PCR results for haemoplasma species using both conventional and quantitative (q) PCR assays.

PCR assay/haemoplasma species found	Number of haemoplasma positives samples (%)	95% confidence intervals
Generic haemoplasma conventional PCR	80/451 (17.7%)	14.2 –21.3%
Haemoplasma species-specific qPCR	60/432 (13.8%)	10.2–16.4%
Any Mhf	48/432 (11.1%)^a^	7.8–13.5%
Any CMhm	60/432 (13.8%)^a^	10.2–16.4%
Any CMt	19/432 (4.4%)^b^	2.4–6.1%
Mhf alone	0	0
CMhm alone	10/432 (2.3%)	0.9–3.6%
CMt alone	0	0
Mhf and CMhm	31/432 (7.1%)	4.5–9.2%
CMhm and CMt	2/432 (0.4%)	− 0.02–1.1%
Mhf and CMt	0	0
Mhf and CMhm and CMt	17/432 (3.9%)	2–5.5%

Mhf = *Mycoplasma haemofelis*.

CMhm = “*Candidatus* Mycoplasma haemominutum”.

CMt = “*Candidatus* Mycoplasma turicensis”.

a No significant difference (p = 0.2) present between the groups labelled with this suffix.

b Significant difference (p = 0.0002) present between this group and those with different suffix.

There was a statistically significant relationship between the haemoplasma species-specific qPCR result and gender. Male cats had a significantly higher overall haemoplasma infection prevalence (p = 0.01), as well as being more likely to be Mhf positive (p = 0.004), CMhm positive (p = 0.009) and CMt positive (p = 0.03), than female cats.

**Fig. 1:**
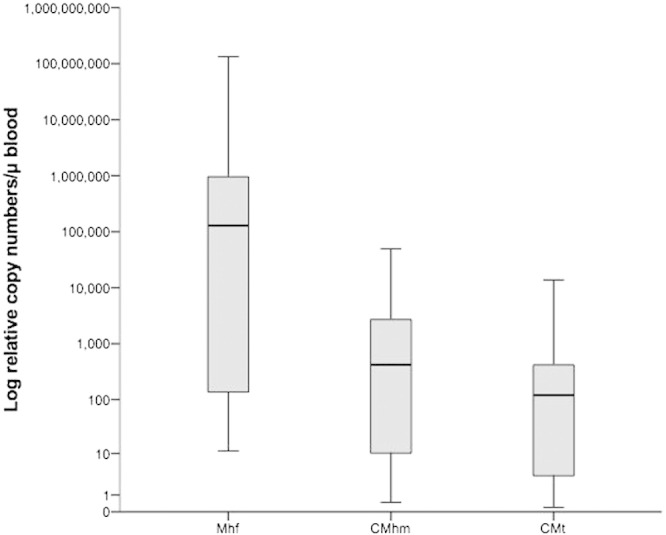
Haemoplasma DNA relative copy numbers/μl blood for each haemoplasma species from samples generating positive results. Boxes represent the 25th, 50th (median) and 75th quartiles with whiskers extending to the greatest and smallest values. Mhf = cats qPCR positive for *Mycoplasma haemofelis*, CMhm = cats qPCR positive for “*Candidatus* Mycoplasma haemominutum”, CMt = cats qPCR positive for “*Candidatus* Mycoplasma turicensis”.

A wide range of relative copy numbers was found for all three haemoplasma species: Mhf-infected cats had relative copy numbers ranging from 0.2 × 10^0^ to 2.54 × 10^8^ relative copies/μl of blood, CMhm-infected cats from 0.2 × 10^0^ to 2.32 × 10^8^/μl and CMt-infected cats from 0.2 × 10^0^ to 1.38 × 10^8^/μl (Fig. 1).

Haematological data (PCV, RBC, haemoglobin, MCV and MCHC) were available for 420 cats (Table 3) and all variables were found to be non-normally distributed. No significant difference in haematological variables was found among the cats grouped according to haemoplasma status (Table 3). No significant correlation was found between haemoglobin concentration and the relative copy numbers of Mhf (n = 45, r_s_ = 0.127, p = 0.4), CMhm (n = 57, r_s_ = 0.246, p = 0.065) or CMt (n = 17, r_s_ = -0.032, p = 0.903), although significance was approached for CMhm. Correlation data between PCV and the relative copy numbers for Mhf (n = 45, r_s_ = 0.031, p = 0.837), CMhm (n = 57, r_s_ = 0.294, p = 0.027) or CMt (n = 17, r_s_ = − 0.101, p = 0.7) revealed a significant correlation for CMhm only, as did correlation data for RBC (Mhf [n = 45, r_s_ = 0.048, p = 0.752], CMhm [n = 57, r_s_ = 0.276, p = 0.038] or CMt [n = 17, r_s_ = − 0.032, p = 0.9]). Due to the small number of CMhm and CMt co-infected cats, no statistical analysis of this haemoplasma status group was possible.

**Table 3:** Descriptive statistics (median and range) and Kruskal–Wallis test results (p value) for haematological variables in cats grouped according to haemoplasma status using species-specific quantitative PCR results.

Variable	Negative cats (n = 372)	CMhm alone (n = 10)	Mhf and CMhm (n = 31)	CMhm and CMt (n = 2)^a^	Mhf and CMhm and CMt (n = 17)	p value
PCV (%)	31 (4–53)	31 (21–38)	29.5 (8–44)	30 (27–34)	27 (12–34)	0.11
RBC (× 10^6^/μL)	8 (0.9–16)	9 (4.4–10.6)	7.5 (1.2–12.5)	8.9 (7–10.7)	8.1 (3–9.8)	0.25
Haemoglobin concentration (g/dL)	11.5 (1.5–19.4)	11.2 (7.8–14.9)	11.5 (2.6–17.2)	11 (10.3–11.8)	10.9 (3.9–15.6)	0.73
MCV (fl)	38 (16–110)	37.4 (30.5–47.4)	38.4 (29.4–66.1)	34.8 (31.5–38.2)	37.9 (27.6–66.9)	0.5
MCHC (%)	36.7 (12.6–71.6)	36.8 (30–51.3)	36.2 (28–55.4)	36.4 (34.7–38.1)	38 (28.8–51–8)	0.74

Values indicate median and range.

PCV = packed cell volume.

RBCs = red blood cells.

MCV = mean corpuscular volume.

MCHC = mean corpuscular haemoglobin concentration.

CMhm = “*Candidatus* Mycoplasma haemominutum”.

Mhf = *Mycoplasma haemofelis*.

CMt = “*Candidatus* Mycoplasma turicensis”.

a Group not included in the statistical evaluation due to the small number of cats.

All 6 haemoplasma-specific primer pairs evaluated for conventional 16S rRNA gene PCR and sequencing amplified the predicted size PCR product. The primer pairs selected for amplifying and sequencing each haemoplasma species (Table 1) were chosen due to their absence of non-target haemoplasma species amplification and a lack of primer dimer formation.

Comparison of nucleotide identity within the 10 Mhf, 10 CMhm and 11 CMt sequences generated in the current study showed > 99.8%, > 98.5% and > 98.8% identity, respectively, with each other. Alignment of the sequences generated in this study and those available on GenBank, for each of the three haemoplasma species, revealed 99.2%–100% identity for Mhf, 98.4%–100% for CMhm and 97.8%–100% identify for CMt.

**Fig. 2:**
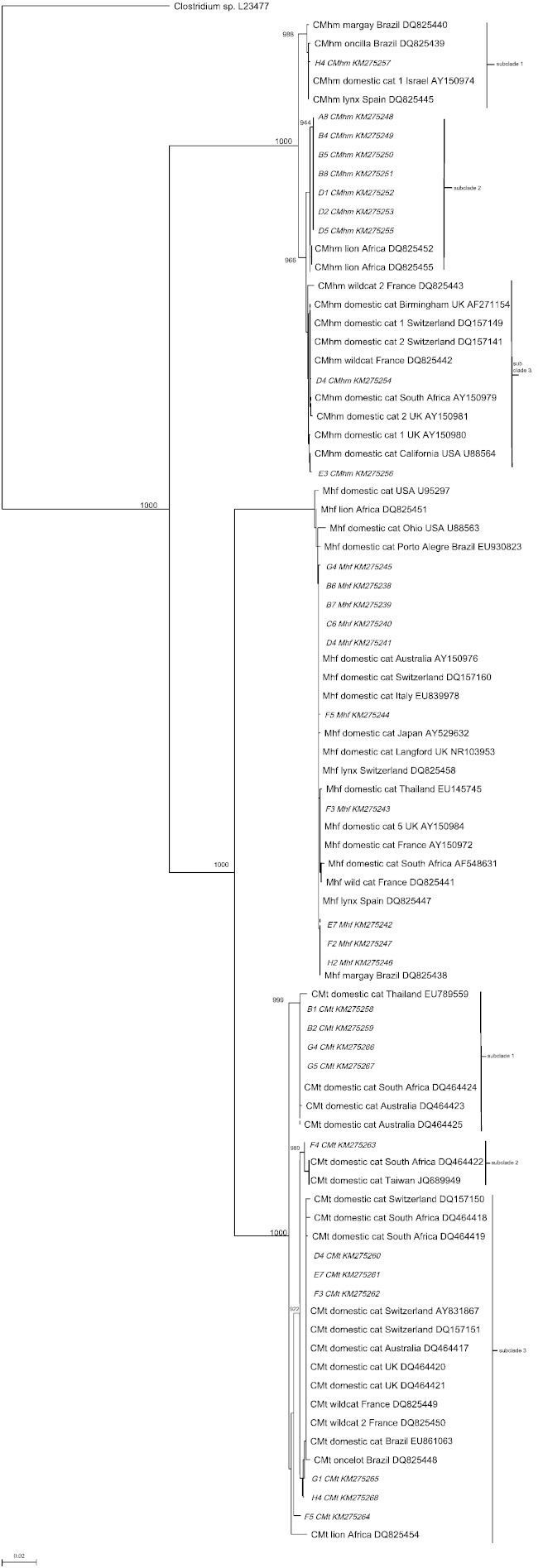
Phylogenetic analysis of near complete 16S rRNA gene sequences from Mhf, CMhm and CMt species in cats from Brasília and surrounding areas. The Neighbor-joining method was used to construct the phylogenetic tree with bootstrap values provided at the nodes (only values ≥ 700 are shown). Evolutionary distances are to the scales shown. *Clostridium* spp. was used as an out-group. GenBank accession numbers are indicated in the figure. Mhf sequences (B6, B7, C6, D4, E7, F2, F3, F5, G4, H2), CMhm sequences (A8, B4, B5, B8, D1, D2, D4, D5, E3, H4) and CMt sequences (B1, B2, D4, E7, F3, F4, F5, G1, G4, G5, H4) were generated in the current study. Mhf = *Mycoplasma haemofelis*, CMhm = “*Candidatus* Mycoplasma haemominutum”, CMt = “*Candidatus* Mycoplasma turicensis”.

The phylogenetic relationships of the sequences generated in this study and those available on GenBank are shown in Fig. 2. This analysis yielded the expected separation of Mhf, CMhm and CMt sequences into three distinct clades accompanied by high bootstrap values. The Mhf sequences all grouped into one single clade with other worldwide Mhf sequences from both domestic and wild cats. However, grouping of the CMhm sequences into three distinct subclades was seen; subclades one and two compromised eight of the ten CMhm sequences from this study and were most closely related to wild cat haemoplasma sequences from Africa, Brazil and Spain; and subclade three consisted of two sequences that shared a closer evolutionary relationship with sequences from domestic cats in Europe (UK and Switzerland). The CMt phylogenetic analysis also showed division into three subclades; five of the 11 CMt sequences from this study grouped into subclades one and two, which were more closely related to sequences from domestic cats in Australia and Africa, whereas the CMt sequences in subclade three were more closely related to domestic and wild cat sequences from European countries (Switzerland, UK, France).

## 4 Discussion

Evidence for the presence of all three feline haemoplasma species in domestic cats in Brazil's capital and surrounding areas was found in this study. The overall haemoplasma prevalence was similar to those reported in the Brazilian states of RJ (Macieira et al., 2008), MA (Braga et al., 2012), SP (de Bortoli et al., 2012), MT (Miceli et al., 2013) and in studies from UK (Tasker et al., 2003a) and Italy (Gentilini et al., 2009), however studies conducted in stray cats from other Brazilian states (André et al., 2014, Santis et al., 2014, Santos et al., 2009), Portugal (Martínez-Díaz et al., 2013) and Italy (Spada et al., 2014) reported higher overall prevalences. The individual CMhm and CMt prevalences were similar to previous Brazilian studies (André et al., 2014, Braga et al., 2012, Macieira et al., 2008, Miceli et al., 2013, Santis et al., 2014, Santos et al., 2009), however the Mhf prevalence was higher than those previously reported in RS (Santos et al., 2009), MA (Braga et al., 2012) and MT (Miceli et al., 2013). As would be expected, the individual prevalence figures found in our study were lower than those in international studies where only cats attending veterinary practitioners (Jenkins et al., 2013, Lobetti and Lappin, 2012, Spada et al., 2014, Sykes et al., 2007) or cats with a clinical suspicion of haemoplasmosis (Jenkins et al., 2013, Lobetti and Lappin, 2012, Spada et al., 2014, Sykes et al., 2007) were sampled; but higher than those found in clinically normal cats used as blood donors (Hackett et al., 2006).

These differences in prevalence may reflect variations in the groups of cats studied and the risk factors for haemoplasma infection. The cat populations from previous Brazilian studies were diverse and included feral free-roaming cats (André et al., 2014, Santis et al., 2014), owned cats from neutering programmes (de Bortoli et al., 2012, Santis et al., 2014), cats attending a feline only clinic and local veterinary hospitals (Macieira et al., 2008, Santos et al., 2009), owned cats screened for blood donation (Santos et al., 2009), feral cats housed in animal shelters (Miceli et al., 2013, Santos et al., 2009) and owned cats with outdoor access (Braga et al., 2012). Direct contact with stray or feral cats in an outdoor environment is believed to be a risk factor for haemoplasma infection (Willi et al., 2006a). Feral cats are probably more likely to be directly infected with haemoplasmas due to fighting, however the number of feral cats included in our study was low, representing 9.3% of the study population and status regarding outdoor access wasn't available for the household cats. In addition, it might be expected that more haemoplasma infection would be detected among cats that attend clinics and veterinary hospitals due to their ill-health. Also, regional differences such as climate and, therefore, the extent and timing of ectoparasite exposure may play a role in haemoplasma prevalence (Willi et al., 2006a). Direct comparison between studies is difficult when there are differences in factors such as inclusion criteria, local weather, indoor versus outdoor access as well as the PCR assays used for diagnosis. The current study, to the best of our knowledge, is the first haemoplasma prevalence study in cats from Brazil that has used qPCR for diagnosis.

Over 80% of haemoplasma positive cats (50 out 60) were co-infected with more than one haemoplasma species in the current study. Although many authors have reported the presence of cats with double or triple infection (André et al., 2014, de Bortoli et al., 2012, Santis et al., 2014), the proportion of co-infected cats in this study was significantly higher than previously reported in RS (16%) (Santos et al., 2009), MS (45%) (Santis et al., 2014), RJ (16.6%) (Macieira et al., 2008), MA (20.8%) (Braga et al., 2012), SP (33.3%) (André et al., 2014, de Bortoli et al., 2012) and MT (6.6%) (Miceli et al., 2013). The presence of many triple and dual co-infected cats in our study could be because the haemoplasma species share related risk factors and/or similar routes of transmission. In line with this is the fact that cats infected with one haemoplasma can also exhibit an increased susceptibility to infection with other haemoplasmas species (Willi et al., 2006a). Additionally, correlations between CMt and Mhf and between CMt and CMhm have been previously reported (Willi et al., 2006b).

Only one cat was positive by the generic haemoplasma PCR but negative by the haemoplasma species-specific qPCRs. This sample may have had a low haemoplasma copy number, below the limit of the qPCR assay sensitivity (Sykes et al., 2007) or it may have contained a novel haemoplasma species with mutations in the qPCR primer or probe binding sites. A false positive result in the generic haemoplasma PCR cannot be ruled out, although negative controls were appropriately negative throughout. Ideally this DNA sample would have been subjected to PCR amplification with an alternative generic haemoplasma qPCR assay (Tasker et al., 2010) and attempts made to sequence any amplicons, but sadly no remaining DNA was available from this cat.

Previous risk factor studies (Jenkins et al., 2013, Tasker et al., 2003a, Willi et al., 2006a) found that male cats are more likely to be infected with haemoplasmas than female cats, in line with the findings of our study. The increased prevalence in males may be because of a higher likelihood of male cats being infected with haemoplasmas due their behaviour patterns such as roaming, biting and fighting. Indeed, it is possible that haemoplasmas are directly transmitted, as suggested by the detection of CMhm and CMt DNA in the saliva of infected cats (Dean et al., 2008, Museux et al., 2009), although aggressive interaction is believed to be required, as direct transmission of haemoplasma infection was found to require exposure to haemoplasma infected blood rather than haemoplasma infected saliva (Museux et al., 2009). Also, other pathogens directly transmitted through fighting interactions, such as feline immunodeficiency virus (FIV), may induce immunosuppression that favours haemoplasma infection (Jenkins et al., 2013). Unfortunately evaluation of the concurrent retrovirus status of all of the cats in this study was not possible.

It has been stated that haemoplasma positive cats are more likely to be anaemic than negative cats (Lobetti and Tasker, 2004, Tasker et al., 2004, Tasker et al., 2009, Willi et al., 2005), mainly during Mhf infection (Gentilini et al., 2009, Jenkins et al., 2013, Lobetti and Lappin, 2012, Lobetti and Tasker, 2004). However, our study as well as others (Barker et al., 2010, Bauer et al., 2008, Macieira et al., 2008, Martínez-Díaz et al., 2013, Spada et al., 2014, Wengi et al., 2008, Willi et al., 2006a) failed to demonstrate association between haemoplasma infection and anaemia. As hypothesised previously (Sykes, 2010, Willi et al., 2006a), changes in haematological parameters during haemoplasma infection vary according to infection stage (acute versus chronic), which haemoplasma species are present, varying pathogenicity, haemoplasma species strain variation and/or host factors such as age. The infection stage for the cats in our study was unknown as this is difficult to determine in naturally infected cats, and age was not recorded.

Some have suggested that anaemia tends to be present in co-infected cats as an additive effect of feline haemoplasma species on clinical disease (Willi et al., 2006b). However, the group of co-infected cats in our study didn't have significantly lower haematological parameters than those with single infections or no infection (Table 3), similar to previous findings (Willi et al., 2006a). Haematological changes in co-infected cats could also be influenced by the same factors (e.g. infection stage) as those proposed for single infections above.

As only naturally infected cats were sampled in the current study, and many cats were co-infected with more than one haemoplasma species, it was not possible to assess which haemoplasma species, if any, infected the cat first. It thus was not feasible to evaluate the effect that one haemoplasma species had had on the presence of other species, and how the order of infection could have affected haematological parameters and relative copy numbers.

Cats with dual or triple haemoplasma co-infections were very common in our study, precluding the use of universal 16S rRNA gene primers for amplification and sequencing of the near-complete 16S rRNA gene of the individual haemoplasma species in these cats. Therefore, the design of new species-specific 16S rRNA gene primers made possible the sequencing of individual haemoplasma species from 12 cats with triple and 10 cats with dual infections (Fig. 2). To the best of our knowledge this is the first report of the use of this approach to obtain individual haemoplasma species sequences from cats with dual and triple co-infections.

Phylogenetic 16S rRNA gene-based analysis showed our sequences to separate into three distinct groups, one for each of the three haemoplasma species (Fig. 2). The CMhm and CMt groups showed further subdivision into subclades as described in some (Tasker et al., 2003b, Willi et al., 2006b), but not all (Braga et al., 2012, Miceli et al., 2013, Santis et al., 2014), previous studies. Interestingly, the CMhm sequences that grouped into subclade two (Fig. 2) in the current study were closely related to African lions, which were also shown to have a high frequency of haemoplasma co-infections in a previous study (Willi et al., 2007). However not all of CMhm sequences from subclade two were derived from cats with multiple haemoplasma infections, as 2 of the 7 cats had single CMhm infections. Thus phylogenetic grouping is not strictly associated with haemoplasma co-infections. The subclade formation within the CMhm and CMt clades showed grouping of sequences from different regions worldwide and from different host types (i.e. wild versus domestic cats). As the phylogenetic divergence among strains may be reflected in phenotypic differences (Tasker et al., 2001), our findings suggest the existence of distinct strains, which may be related to varying pathogenicity, geographical distribution or host species. Unfortunately no specific definitions of exactly what constitutes a haemoplasma species strain exist.

Unlike the CMhm and CMt sequences, and as previously observed (Braga et al., 2012, Tasker et al., 2003b, Willi et al., 2007), the Mhf sequences in the current study did not reveal any subclade formation. The reasons for this difference in the 16S rRNA gene stability among haemoplasma species are not known.

Although phylogenetic information derived from a highly conserved gene, such as 16S rRNA, is often used, additional phylogeny using non-16S rRNA gene sequences, as has been undertaken in a few studies (Hicks et al., 2014, Peters et al., 2008a, Tasker et al., 2003b), would have been helpful to support our conclusions. However, the high number of co-infected cats made the amplification of other genes, such as RNase P RNA gene, very difficult since the design of species-specific primers is fraught with difficulty due to the short length of these genes. Further analysis of a larger number of genes and cases in the future, using alternative methods, would however be helpful in further refining the relationships established in our phylogenetic study.

## 5 Conclusion

This represents the first study to molecularly characterise the near-complete 16S rRNA gene sequences in haemoplasmas from domestic cats with a high prevalence of co-infections, using a technique employing species-specific primers. Haematological variables were also evaluated. Future studies should investigate the significance of co-infections in wild and domestic hosts within the epidemiology of haemoplasma infections.
